# Decision support by machine learning systems for acute management of severely injured patients: A systematic review

**DOI:** 10.3389/fsurg.2022.924810

**Published:** 2022-10-10

**Authors:** David Baur, Tobias Gehlen, Julian Scherer, David Alexander Back, Serafeim Tsitsilonis, Koroush Kabir, Georg Osterhoff

**Affiliations:** ^1^ Department for Orthopedics and Traumatology, University Hospital Leipzig, Leipzig, Germany; ^2^ Center for Musculoskeletal Surgery, Charité University Medicine Berlin, Berlin, Germany; ^3^ Clinic for Traumatology, University Hospital Zurich, Zurich, Switzerland; ^4^ Clinic for Traumatology and Orthopedics, Bundeswehr Hospital Berlin, Berlin, Germany; ^5^ Department of Orthopaedics and Trauma Surgery, University Hospital Bonn, Bonn, Germany; ^6^ Department for Orthopedics, Traumatology and Plastic Surgery, University Hospital Leipzig, Leipzig, Germany

**Keywords:** trauma, polytrauma, decision support, machine learning, deep learning, artificial intelligence, neural networks, prediction

## Abstract

**Introduction:**

Treating severely injured patients requires numerous critical decisions within short intervals in a highly complex situation. The coordination of a trauma team in this setting has been shown to be associated with multiple procedural errors, even of experienced care teams. Machine learning (ML) is an approach that estimates outcomes based on past experiences and data patterns using a computer-generated algorithm. This systematic review aimed to summarize the existing literature on the value of ML for the initial management of severely injured patients.

**Methods:**

We conducted a systematic review of the literature with the goal of finding all articles describing the use of ML systems in the context of acute management of severely injured patients. MESH search of Pubmed/Medline and Web of Science was conducted. Studies including fewer than 10 patients were excluded. Studies were divided into the following main prediction groups: (1) injury pattern, (2) hemorrhage/need for transfusion, (3) emergency intervention, (4) ICU/length of hospital stay, and (5) mortality.

**Results:**

Thirty-six articles met the inclusion criteria; among these were two prospective and thirty-four retrospective case series. Publication dates ranged from 2000 to 2020 and included 32 different first authors. A total of 18,586,929 patients were included in the prediction models. Mortality was the most represented main prediction group (*n* = 19). ML models used were artificial neural network ( *n* = 15), singular vector machine (*n* = 3), Bayesian network (*n* = 7), random forest (*n* = 6), natural language processing (*n* = 2), stacked ensemble classifier [SuperLearner (SL), *n* = 3], k-nearest neighbor (*n* = 1), belief system (*n* = 1), and sequential minimal optimization (*n* = 2) models. Thirty articles assessed results as positive, five showed moderate results, and one article described negative results to their implementation of the respective prediction model.

**Conclusions:**

While the majority of articles show a generally positive result with high accuracy and precision, there are several requirements that need to be met to make the implementation of such models in daily clinical work possible. Furthermore, experience in dealing with on-site implementation and more clinical trials are necessary before the implementation of ML techniques in clinical care can become a reality.

## Introduction

Time is considered one of the significant factors for patient outcomes after major trauma. Depending on injury severity, a rapid medical assessment, life-saving on-site treatment, and transportation to an appropriate trauma center are essential to improve survival rates. Therefore, constant improvement in prehospital settings in resuscitation, rapid transit, and adequate initial treatment in hospitals have a substantial impact on survival rates (1).

The introduction of standardized training and education programs has improved the quality of care for severely injured trauma patients—both in the preclinical field and in the emergency trauma room. An example is Advanced Trauma Life Support. Altogether, educational training and standards have led to improvements in the factor of time and treatment quality (2).

Although emergency care and surgical care improvement led to a better outcome, up to 8.0% of all trauma patients’ death are considered preventable or potentially preventable (3). Management errors arise because of time pressure, inexperience, reliance on memory, multitasking, information flow analysis, and failures in trauma team coordination, particularly during the initial minutes of patient reception and resuscitation in emergency rooms. Even in established trauma centers with experienced trauma care professionals, despite guidelines, protocols, and continuous performance improvement, protocol compliance was only 53% (4).

In the age of digitalization, connecting computer-generated prompts through visual and auditory displays within the resuscitation may enhance trauma care professionals’ interaction and reduce errors of omission and miscommunication. In addition, the past decade led to the excitement for the potential to apply deep-learning algorithms to healthcare. This subtype of artificial intelligence (AI) has the ability to improve the accuracy and speed of interpreting large datasets, such as images, speech, and text (5). Machine learning (ML) deals with the estimation of outcomes based on past experiences and data patterns using a computer-generated algorithm (6).

This systematic review aims to evaluate the existing literature on how ML can change the decision support of acute management in severely injured patients.

## Materials and methods

### Study design

A systematic review of the literature according to the PRISMA (Preferred Reporting Items for Systematic Reviews and Meta-analyses) checklist and algorithm was conducted, with the goal of finding all articles describing the value of machine learning systems in the context of acute management of severely injured patients (7).

### Study characteristics

Investigations between 2000 and January 2021 were included. For analysis, prospective and retrospective observational investigations including database studies were considered.

### Information source

The authors performed a systematic search of the PubMed/Medline and Web of Science (Core Collection) databases for eligible investigations.

### Search

The search terms were (trauma) AND ((decision) OR (predict*) OR (assist*)) AND ((artificial intelligence) OR (neural network) OR (machine learning) OR (deep learning)).

### Study selection

**Figure 1:**
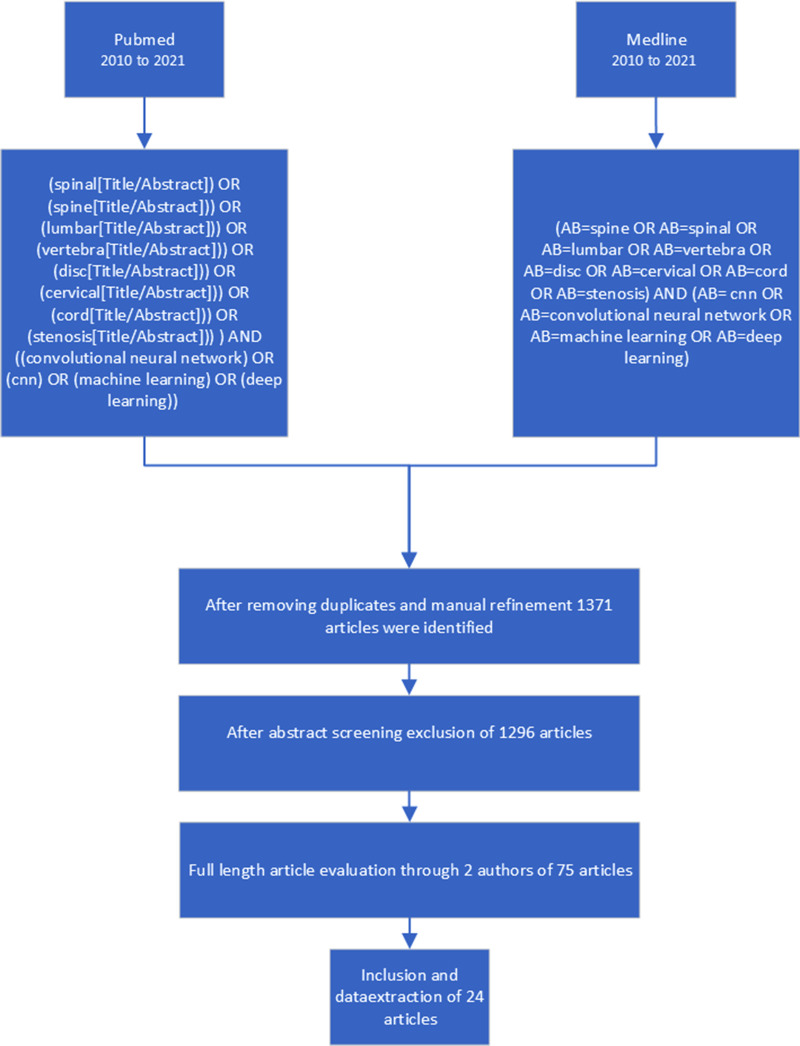
Selection process for the systematic review - flow chart.

The authors limited the research to observational studies, while systematic reviews, meta-analyses, case series, and case reports were excluded. Titles and abstracts were reviewed after the removal of duplicates. The remaining full texts were checked for suitability by all authors, and disagreement was resolved by consensus. In cases of doubt, articles were included in the next stage. A flowchart of the filtering stages (titles, abstracts, full-length texts) is shown in Figure 1.

### Data items

Studies were selected according to the following inclusion criteria: (a) case series, cohort studies, clinical trials, or registry data studies regarding decision support by self-learning systems for the acute management of adult civilian trauma patients, (b) studies that used parameters available during initial assessment and resuscitation in the trauma bay, (c) studies that used at least one physiologic parameter (e.g., heart rate), (d) models that predicted patient-related outcome (e.g., mortality, hemorrhage, need for emergency intervention), and (e) articles published in English or German language.

Exclusion criteria are as follows: (a) case reports or case series with fewer than 10 patients, (b) review articles, (c) animal studies, (d) cadaver studies, (e) studies including only patients with isolated non-life-threatening injuries, (f) studies including only patients with isolated traumatic brain injury, (g) models relying on imaging data, and (h) studies that predicted an effect that would become immanent after more than 30 days (e.g., 1-year survival).

### Synthesis of results

We extracted data concerning study characteristics including author names, title, year of publication, journal of publication, number of patients, time of follow-up, and type of study. For the description of the study population, the number of patients and age were collected. Outcome parameters were analyzed according to the inclusion criteria and were assigned to five predicted outcomes: (1) injury pattern, (2) hemorrhage/need for transfusion, (3) emergency intervention, (4) ICU/length of hospital stay, and (5) mortality.

For all included studies, we used the Oxford Centre for Evidence-Based Medicine 2011 to define the level of evidence (OCEBM Levels of Evidence Working Group 2011).

## Results

Thirty-six articles met the inclusion criteria; among these were 2 prospective and 34 retrospective case series. Publication dates ranged from 2000 to 2020 and included 32 different first authors. A total of 18,586,929 patients were included in the prediction models. Machine learning models used were artificial neural network (ANN; *n* = 15), singular vector machine (*n* = 3), Bayesian network (*n* = 7), random forest (*n* = 6), natural language processing (*n* = 2), stacked ensemble classifier (SL, *n* = 3), k-nearest neighbor (KNN, *n* = 1), belief system (*n* = 1), and sequential minimal optimization (*n* = 2) models. Thirty articles assessed results as positive, five showed moderate results, and only one article described negative results to their implementation of the prediction model (Table 1). The quality of included articles showed OCEBM Levels of Evidence of 3, correlating with the retrospective character of model training.

**Table 1:** Overview of the 36 included studies, sorted by name and prediction groups.

Year	Author	Year	Input	Main prediction group	Model type	Model description	Patient type	No. of patients	Outcome compared to other predictions	Outcome parameter	Result	Conclusion
2020	Ahmed	2020	Vital signs	Mortality	Deep FLAIM	Biostatically rigorous two-phase ML framework	Trauma ICU patients	3,014	Other ML models	AUC, Sens, ACC	Positive	Very positive, the model outperforms other machine learning models
2019	Almagrabi	2019	Vital signs	Mortality	Belief System	MAKER RIMER combination	Trauma Audit and Research Network database	177,014	Other ML models	AUC	Positive	Positive, with an AUC of 0.683 for mortality
2019	Christie	2018	Patient histories from 3 hospital sites	Mortality	SL	Ensemble machine learning algorithm	Trauma patients	28,212	Standard scoring methods	AUC	Positive	Very positive prediction of mortality (AUC range: 0.90–0.94)
2018	Chrisite	2019	Multiple inputs	Mortality	SL	Ensemble machine learning algorithm	Critically injured database prospective cohort data	1,494	N/A	AUC	Positive	Good prediction for mortality (AUC range: 0.94–097), multiorgan failure (AUC range: 0.84–0.90), and transfusion (AUC range: 0.87–0.90).
2000	DiRusso	2000	Prehospital variables, ER admission variables, ISS	Mortality	ANN	Standard feed-forward back-propagation neural network	Regional area trauma system	10,609	TRISS, ISS	ROC	Positive	Compared to TRISS and ISS improvement in ROC (0.912). ANN suited for implementation
2005	Fuller	2005	ISS, TS, age, sex, mode of injury, others	Mortality	ANN	Back-propagation neural network	Trauma register	2,510	TRISS	Relative error	Positive	The ANN model was able to predict mortality with a relative error of 5% and was superior to TRISS (relative error 27.7%
2018	Kim	2018	National sample project dataset, wearable device data	Mortality	ANN, others	Logistic regression, random forest, deep neural network	Blunt and penetrating injuries	460,865	GCS	AUC	Positive	Of the tested models, all alternatives to GCS showed superior AUC, with the proposed ANN showing the best results (AUC: 0.89)
2019	Gorcyca	2019	TRISS variables, ICD 9	Mortality	TSM	Logistic regression, random forest, gradient boost machine, feed-forward neural network	Trauma patient database	1,432,5172	Bayesian network, injury severity score, Harborview assessment of risk of mortality, trauma mortality prediction model	ACC, F-score	Positive	The model showed superior predictive power compared to other models
2008	Pearl	2006	Multiple inputs	Mortality	ANN	Back-propagation, feed-forward ANN	Database	1,433,024	GCS	ROC	Positive	Improved predictive power compared to GCS
2006	Pearl	2008	Multiple inputs	Mortality	ANN	Back-propagation, feed-forward ANN	Swedish trauma registry	7,688	GCS	ROC	Positive	Implemented models performed well; however, additional variables could improve the performance of the ANN
2019	Rau	2019	Multiple inputs	Mortality	ANN (Stuttgart neural network Simulator)	Back-propagation, feed-forward ANN	Trauma patients	18,811	TRISS, SVM, LR	ACC, Sens	Positive	Implemented ANN showed the highest score in balanced accuracy (75.1%) compared to the other prediction models
2017	Roveda	2017	Multiple inputs	Mortality	ANN, naive Bayes, logistic regression, random forest	Two hidden layers, 5/8 hidden neurons per layer depending on prediction	CRASH II patients	20,207	ANN, naive bayes, logistic regression, random forest	ROC	Moderate	Promising results, but the used dataset was limited in dimensionality of the given patient data
2017	Schetinin	2013	Multiple inputs	Mortality	Bayesian network	Bayesian averaging over decision tree model	NTDB database	571,148	TRISS	Sens, spec	Positive	When compared to TRISS classification accuracy showed significant improvement
2013	Schetinin	2017	Multiple inputs	Mortality	Bayesian network	Bayesian averaging over decision tree model	NTDB database	14,840	TRISS	AUC	Positive	The proposed network outperformed the standard methods in predictive performance
2017	Sefrioui	2017	Seventeen features	Mortality	Random forest, ANN, LR, KNN, naive Bayes, PART division lists, SMO	Multiple models for mortality on a big data set	NTDB database	656,092	TRISS	ACC	Positive	From the tested models, the random forest model showed highest accuracy and improvement over TRISS for mortality prediction
2020	Servia	2020	Multiple inputs, pre- and postresuscitation	Mortality	Logistic regression, ANN, SMO, Bayesian networks, AdaBoost, Bagging, others	Multiple models for mortality on a big data set	RETRAUCI database	9,625	All implemented model types	F-Score, AUC	Positive	Authors implemented a range of different models, fittest best for the task of mortality prediction were SMO and Bayesian network model
2020	Tsiklidis	2020	32; 8 after reducing variables	Mortality	Estimator gradient boosting classifier	Estimator gradient boosting classifier	NTDB Database	799,233	N/A	AUC	Positive	The proposed model was able to predict mortality correctly in 92.4% of cases
2005	Wolfe	2006	Multiple features, prehospital	Mortality	ANN, logistic regression, classification tree	Three different investigators on the same database	Patients with blunt trauma Victorian state trauma registry	4,014	N/A	Sens, spec	Negative	None of the implemented methods were optimal in predicting mortality but useful insides for further developments were collected
2015	Gholipour	2015	TRISS variables	ICU LOS, mortality	ANN	Standard feed-forward back-propagation neural network	Trauma patients admitted to ER	125	N/A	Sens, spec	Positive	Positive outcome with no significant difference of ICU LOS predictions and real LOS. High sensitivity for mortality
2020	Bhat	2020	BGA parameters	Hemorrhage/transfusion	SVM, bagged decision tree, ANN with a Bayesian regulator	Different models were compared in their ability to classify similar to HISS	100 cases, data synthetically derived (SFRP)	100	HISS	ACC, Sens	Positive	All models were highly accurate without significant differences (range ACC 0.91–0.93)
2008	Chen	2008	Vital signs	Hemorrhage/transfusion	Linear classifier	Linear classifier	Helicopter transport trauma patient	898	Schock Index	AUC	Positive	Good prediction with AUC 0,76 for identification of patients in need for transfusion
2002	Clarke	2002	Multiple inputs	Hemorrhage/transfusion, emergency intervention	Trauma AID	Trauma AID, early algorithm	Trauma patients with penetrating thoracoabdominal trauma	97	N/A	N/A	Moderate	Moderate results using the model to decide treatment strategy. Of 40 cases 5 computer predicted strategies were altered
2018	Hodgman	2018	Data from resuscitation bay PROMMTT	Hemorrhage/transfusion	App model (Mina et al.)	N/A	Patients needing at least 1 transfusion at resuscitation bay	1,245	N/A	AUC	Moderate	Moderate predictive performance of the app model for need for massive transfusion (AUC: 0.694)
2020	Li	2020	Vital signs, BGA parameters	Hemorrhage/transfusion	Random forest model	Random forest algorithm based on decorrelated decision trees	Database, ER	1,405	Logistic regression model	F1-Score	Moderate	Superior F1 Score (93.4%) but lower AUC (0.81) when compared to logistic regression model for predicting acute traumatic coagulopathy
2013	Hubbard	2013	Data from resuscitation bay PROMMTT	Hemorrhage/transfusion	SL	Ensemble machine learning algorithm	PROMMTT patients	980	N/A	ROC, AUC	Positive	Superior prediction of ongoing transfusion with SL over naive approaches for different time intervals
2006	Partridge	2006	16 parameters, resuscitation bay parameters minus BGA	Hemorrhage/transfusion	Bayesian network	Decision tree with Bayesian averaging	Trauma patients’ “difficult” cases	316	Maximum a posteriori	N/A	Positive	Early work on decision tree implementation with positive results compared to other implementations
2005	Walczak	2005	17 features	Hemorrhage/transfusion	ANN	Back-propagation	Patients trauma	1016	N/A	Sens, spev, mean absolute difference	Positive	The authors demonstrated high sensitivity and specificity for predicting transfusion indication in trauma patients with the implementation of ANN
2017	Gu	2017	Speech input	Injury pattern	NLP	CNN, LSTM	Thirty local cases	30	N/A	ACC	Moderate	Authors could show a 79.12% accuracy for phase prediction
2020	Kulshrestha	2021	Text-based inputs	Injury pattern	NLP	Logistic regression with elastic net regularization	Patients local hospital with thorax injury	6,891	N/A	AUC	Positive	Authors were able to show good prediction of differentiation between severe and non-severe injury to the thorax (AUC 0.88)
2015	Metzger	2015	Lab parameters, comorbidities, demographic	Injury pattern	“Figaro” model	Stacked ensemble classifier (similar to SL)	Trauma patients’ subsection vascular injuries	2,643	Other classifiers	Sens, acc	Positive	Good prediction for death, multi-organ failure and transfusion. Superior when compared to other classifiers
2002	Ogunyemi	2002	Resuscitation bay parameters, clinical findings	Injury pattern	Trauma SCAN, Bayesian network	Trauma SCAN, Bayesian network	Patients with gunshot wounds chest and abdomen	26	Trauma SCAN diagnosis, vs. Trauma SCAN and clinical findings in a Bayesian network	AUC	Positive	Implementing geometric and probabilistic reasoning showed an improvement of prediction for TraumaScan
2020	Paydar	2020	Multiple inputs	Injury pattern	Bagged, SVM, KNN, AdaBoost, neural network	Different classifiers compared	Trauma patients >16 years	1,107	Bagged best, SVM second, KNN, Adaboost, neural network	ACC	Positive	Authors were able to predict deterioration of injured patients with high accuracy, through bagging and SVM models (99%)
2019	Harvin	2019	Multiple inputs	Emergency intervention	Random forest algorithm	Random forest algorithm based on decorrelated decision trees	Patients admitted to trauma center	25,983	N/A	ACC	Positive	Good prediction of damage control laparotomy (ACC range 0.772–0.857)
2002	Hirshberg	2002	Bullet trajectory, admission systolic blood pressure	Emergency intervention	ANN	Array of ANNs, two hidden layers, logistic activation, back-propagation	Patients with single abdominal gunshot wound	312	N/A	Classification rate, ACC, sens, spec	Positive	The authors were able to achieve excellent correct classification rate (0.96) and AUC (0.94)
2014	Liu	2014	Vital signs, monitoring, GCS	Emergency intervention	ANN	Multilayer perceptron	Patients transported *via* helicopter with trauma	104	Multivariate logistic regression	ROC, AUC	Positive	Compared to multivariate logistic regression models, the ANN showed higher AUC (0.99) in prediction performance for emergency intervention
2014	Liu	2014	Feature sets, patient record	Emergency intervention	ANN	Multilayer perceptron and simple rule-based algorithm	Patients transported *via* helicopter with trauma	79	N/A	Mean absolute error	Positive	The authors showed higher ROC with the proposed implementation of ANN for emergency intervention prediction

Deep FLAIM framework, deep Fahad-Liaqat-Ahmad intensive machine framework; ML, machine learning; AUC, area under the curve; Sens, sensitivity; ACC, accuracy; MAKER, maximum likelihood evidential reasoning; RIMER, rule-based inference methodology using the evidential reasoning approach; BGA, blood gas analysis; SVM, support vector machine; ANN, artificial neural network; HISS, hemorrhage intensive severity and survivability score; SFRP, sensible fictitious rationalized patient; SL, “Super Learner”; ER, emergency room; ISS, injury severity score; TRISS, trauma and injury severity score; ROC, receiver operator characteristic; TS, trauma score; ICU LOS, intensive care unit length of stay; spec, specificity; TSM, trauma severity model; PROMMTT, prospective, observational, multicenter, majof trauma transfusion; GCS, Glasgow coma scale; NLP, natural language processing; KNN, k-nearest neighbor; CRASH, Clinical Randomization of an Antifibrinolytic in Significant Hemorrhage; SMO, sequential minimal optimization.

### Predicting injury patterns

Over the past 20 years, various research groups have been working with computer-assisted systems to partially automate the analysis of patient data in the resuscitation room to assess injury patterns. Depending on the study, prehospital and hospital-acquired parameters were used for predicting patients’ injury patterns.

In 2002, Ogunyemi et al. explored the possibility of using probabilistic graphic models in combination with 3D reconstruction to analyze penetrating chest and abdominal trauma with the aim of predicting outcomes based on the location of penetration. The TraumaScan tool developed for this purpose used a combination of the location of the entry wound and the patient’s symptoms and parameters. The use of such software in the treatment of penetrating injury is an effective tool to make the treatment of injured people more time effective and safe (8).

Metzger et al. tested the possibility of using various artificial intelligence (AI) algorithms to detect vascular injury based on the initially collected patients’ parameters to aid in treatment decisions and the identification of critical patients. For this purpose, 2,643 patients were selected and parameters were extracted. The parameters were tested for outcomes on different classifiers and combinations of multiple classifiers. The use of multiple classifiers deployed on these parameters produced the best results (9).

The work of Gu et al. follows a new approach to classifying the individual phases of a trauma resuscitation (pre-arrival, patient arrival, primary survey, secondary survey, and postsecondary survey) and uses the spoken words in the resuscitation room. For this purpose, microphones were installed and the recorded words were converted into individual path-finding phrases. This was tested on 24 recorded trauma resuscitations and converted into an algorithm by deep learning processes. Subsequently, this process was performed on six recorded cases with a matching accuracy of almost 80%. In summary, audio analysis during resuscitation room management shows a novel implementation of data collection and options for phase classification (10).

In the most recent study on classifying injury patterns in polytrauma patients, Paydar et al. showed that early classification of the injury pattern and decision-making support are relevant when the patient prognosis is poor. Using data from 1,107 trauma patients and using various analytics algorithms, they aimed to investigate the benefits of AI-aided decision-making for the treatment of polytrauma patients. For this purpose, paraclinical and clinical data were extracted. Diastolic blood pressure, GCS, and BE after resuscitation crystallized as the most impactful parameters; the outcome was predicted with high accuracy of 0.99 (11).

Kulshrestha et al. retrospectively evaluated data from 6,891 trauma patients and analyzed 450,000 corresponding documents. Using natural language processing, ANN was trained for automated analysis. The authors were able to show that the implementation of natural language processing can aid in the adequate classification of thoracic trauma (12).

### Predicting hemorrhage/need for transfusion

Walczak trained ANN for the prediction of the need for blood transfusions (fresh frozen plasma, packed red blood cells, and platelets) using data from 508 retrospective patients who were transferred to their trauma center between January 1996 and December 1997 using the back-propagation method (13). The input variables for the ANN were easily accessible patient characteristics obtained on admission to the ER, such as sex, age, blood pressure, Glasgow coma scale, and so on. Trauma patients with no transfusions but with similar epidemiological data were added to the data set, which resulted in a total of 1,016 data sets (training set with 538 patients and hold-out-sample with 478 patients). The main finding was that the proposed ANN was able to predict blood transfusions with a mean absolute error (MAE) of 7.02 units for patients who received 0–174 units in total (all types of transfusions within the first 24 h after admission), but the ANN showed better MAE (5.49 units) for specific blood transfusion types in shorter time periods (13).

Chen et al. introduced a classifier as a decision assist tool for identifying hypovolemia in trauma patients being transported from the scene *via* a helicopter when reliable vital parameters are hard to assess (14). The working group used data from 898 trauma patients and included 627 subjects with 71 cases of major bleeding. The ensemble classifier, which was fed with five easily assessable vital parameters every second (RR, RR, DBP, SBP, and SaO_2_) showed an area under the curve (AUC) of almost 0.8 after 14 min of transport for the prediction of life-threatening hemorrhage and was able to tolerate missing variables better than linear classifiers (14).

In the comparison of standard stepwise logistic regression analysis to new SL techniques, the SL showed a superior prediction of mortality in this complex dynamic multivariate data set at several time intervals.

Hodgman et al. utilized the PROMMT database in 2018 for validation of a smartphone app model for predicting the activation of mass transfusion protocol or MT delivery for five different mass transfusion definitions: 10 units of packed red blood cells (PRBCs) within 24 h (1), resuscitation intensity score ≥4 (2), critical administration threshold (3), 4 units of PRBCs within 4 h (4), and 6 units of PRBCs within 6 h (5). Examining 1,245 patients, the smartphone app showed consistent prediction for the need for MT, MTP activation, and MT delivery with AUC ranging from 0.694 to 0.711 regardless of the MT definition (15).

Christie et al. assessed 1,494 severely injured patients with 2,397 variables in the time period from February 2005 to April 2015 for several outcomes including the need for transfusion and need for mass transfusion (>10 L in 24 h) several times after trauma (2–120 h) (16).

SL technique was applied to the data sets, and early blood transfusion was sufficiently predicted with an AUC of 0.82 and 0.84–0.88 for interval prediction throughout the first 72 h after admission (16).

Recently, Bath et al. introduced the hemorrhage intensive severity and survivability score (HISS) using five biomarkers: glucose, lactate, pH, potassium, and oxygen tension. The working group created 100 sensible fictitious rationalized patients (SFRPs) and let five trauma experts rate their HISS score for triage (0 = low, 1 = guarded, 2 = elevated, 3 = high, 4 = severe). Afterward, linear support vector machine, ensemble bagged decision tree, ANN with Bayesian regularization algorithm, and possibility rule-based using function approximation (PRBF) were evaluated for their ability to accurately classify the 100 entries of the SFRP data set with an identified adequate training set of 75, and it was felt that these classification algorithms can be used as an adjunct to the HISS due to an accuracy higher than 91% in the clinical setting (17).

### Predicting the need for emergency intervention

One of the earlier publications covering the use of an AI-based technology was a study by Clarke et al. with the TraumAID computer program in 2002. Here, a retrospective analysis of 97 cases showed a significantly higher evaluation of three raters for TraumAID’s protocols over actual care in 64 cases. TraumaAID was used by residents in 40 cases in the emergency department, and in 5 of these, this resulted in a change of evaluation, diagnosis, or treatment, while none of these changes was judged to be an error by the majority of the raters (18).

In the same year, Hirshberg et al. reported on the creation of an ANN (ANN) for the prediction of damage control operations in patients with a single abdominal gunshot injury. After training the ANN on data of 312 patients, the authors tested it on 34 cases. A sensitivity of 88% and a specificity of 96% were achieved. Variables like systolic pressure, bullet path, or trajectory were determined as strong inputs (19).

Prediction of performing a damage control laparotomy in trauma patients was also the focus of a study by Harvin et al. 2019. In a single center, a quality improvement intervention had been introduced in advance, successfully reducing the rate of damage control laparotomies (DCLs) without increasing morbidity or mortality. A random forest computer learning algorithm, based upon decorrelated decision trees for prediction, was used to analyze 72 variables for their predictive value for a DCL, identifying some significant correlations. The authors concluded that ML could be used to point out the effects of interventions on surgeons’ decision-making (20).

Wolfe et al. created three prediction models for outcomes following nonpenetrating trauma using a data set of 4,014 patients, of which a first subgroup was used for training the model and a second one for external validation. Models based on data from the scene of injury were developed for an intensive care unit (ICU) stay (complete data sets 1,324; 33%) or later death (complete data sets: 2,059; 51.3%). Statistical models used were logistic regression, classification trees, and ANN. None of the models was seen as optimal and met the self-given performance criteria, therefore being the only article in this systematic review showing negative results (21).

Liu et al. published two papers in 2014. In the first one, they described the development and validation of a multiparameter ML algorithm for predicting the need for life-saving interventions in trauma patients. All patients sustained severe blunt and penetrating injuries and were transported to two Level I trauma centers by helicopter. The authors used 79 patient records with over 110,000 feature sets to train the system and applied the trained network to data from 104 patients. The algorithm showed positive results for the prediction of trauma patients needing live-saving interventions (22).

In another paper from the same year, Liu et al. analyzed the data of 104 patients extracting a combination of vital signs, heart rate complexity, and others, again applying ML tools to identify the need for life-saving interventions. They showed that an ML model has superior performance over multivariate logistic regression models (23).

### Predicting admission to ICU/length of hospital stay

For healthcare providers, the assessment of valences in intensive care units and their distribution at any given point in time is essential. In 2015, Gholipour et al. used trauma and injury severity score (TRISS) variables to train a feed-forward propagating ANN to predict the length of stay in the ICU and mortality. For this purpose, data from 95 trauma patients admitted to the emergency room were retrospectively used to train an ANN and tested on 30 other cases.

Overall, the results were good, with a sensitivity of 75% and a specificity of 96% for the survival of trauma patients and no significant difference in the ICU length of stay prediction and real length of stay (24).

### Predicting mortality

In 19 studies, mortality was the most common prediction in this review. A total of 17,972,347 patients were included overall. In total, 13 different ML algorithms and models were considered, showing positive results for 18 of 19 studies. Models based on ANNs were most prevalent in this review, with the adaption of this architecture in 16 papers (84% of mortality predicting articles).

Ahmed et al. identified 3,041 trauma patients admitted to their trauma surgery ICU. Univariate and multivariate analyses on mortality were performed, and ANN and other ML models were deployed on the extracted data. With an accuracy of 92.3% and sensitivity of 79.1%, the ANN-based deep-FLAIM model outperformed other tested methods (25). Almagrabi et al. used the TARN database to access retrospective vital sign data from 177,014 patients to predict mortality, testing different ML models. With AUC values of 0.6882, 0.6829, and 0.678, respectively, logistic regression, interpretable ML, and ANN were the best classifiers tested (26).

An earlier study by DiRusso et al. compared established mortality predictors, namely, TRISS and Injury Severity Score (ISS), with a feed-forward backpropagation neural network, showing its superiority with a receiver under the operating curve (ROC) of 0.912 for the ANN and 0.895 and 0.766 for TRISS and ISS, respectively. Including 10,609 patients admitted to 24 hospitals in a seven-county region, this multicenter study confirms the previous work of the research group (27). A 2005 study from Fuller et al. also compared TRISS predictions with ANN including 2,510 patients from the CAMC Trauma registry. Using ISS and six other inputs, the ANN outperformed TRISS predictions with a relative error of 5% compared to 28%. Unfortunately, no AUC, ROC, accuracy, or sensitivity were calculated in this study (28). A more recent study from 2015 by Gholipour et al. used input variables of TRISS on 125 patients to predict mortality and length of stay, showing satisfactory results with a sensitivity and specificity of 75% and 97%, respectively, for the prediction of mortality by ANN. Comparing the results to TRISS, the sensitivity and specificity of TRISS showed better results with 81% and 95%, respectively (24). In 2019, Gorczyca et al. were able to achieve excellent classification rates for mortality prediction, comparing a state-of-the-art ANN to several other prediction models including Bayesian networks, ISS, and others (29). Hubbard et al. used SL to predict mortality and the need for transfusion within discrete time intervals (30–90, 90–180, and 180–360 min) in patients meeting the criteria for highest-level trauma activation in 10 major Level I hospitals. SL outperformed the standard methods for predicting future mortality, with the greatest difference being the prediction of death at the 180–360 min interval (AUC SL 0.92 vs. 0.55 for standard methods) and a 5% increase compared to logistic regression models in prediction performance (30). Kim et al. extracted 460.865 cases of blunt and penetrating trauma from the National Trauma Data Bank (NTDB) and assessed the implementation of a consciousness index as well as ML algorithms to predict mortality. With an AUC of 0.89, the deep neural network showed the best results in predicting mortality. The used input variables were chosen as though they were collected by wearable patient devices, showcasing the feasibility of such devices (31).

Pearl et al. published two studies in 2006 and 2008 using ANNs with eight and five parameters, respectively, for the prediction of mortality for clinicians and nonclinicians [32]. In both studies, the NTDB data were used, including 1,433,024 patients in 2006 and 7,688 patients in 2008. Pearl et al. could show that ANNs predicted mortality with prehospital variables well, with a correct prediction of 91% in 2006, but could not show improvement when excluding pulse and other input variables in 2008 for nonclinician use of ANNs (33).

In a recent study by Rau et al., the authors compared mortality prediction for 18,811 patients using TRISS, ANN (neural network configured *via* the Stuttgart neural network simulator), support vector machine, and logistic regression. Results showed high accuracy for all four models but the highest specificity (51.5%) for ANN (34). Another retrospective analysis by Roveda et al. included 20,207 patients from the CRASH (Clinical Randomisation of an Antifibrinolytic in Significant Haemorrhage) database to predict mortality, as well as ICU stay and need for surgical intervention. All tested models (logistic regression, Bayesian network, random forest, ANN) showed similar results, concluding that a combination of the above-mentioned models could enhance the performance of predictive power (35).

Schetinin et al. used Bayesian averaging over decision trees to predict the mortality of NTDB data by considering 571,148 cases, comparing results with TRISS estimates, and concluding that the goodness of fit was superior to the TRISS method (36). A study from 2017 by Sefrioui et al. compared different ML approaches to the NTDB database, characterizing every patient (*n* = 656.092) by 17 features, including GCS, vital signs, and other parameters, with TRISS prediction. In their testing, the random forest approach showed the most promising results (ACC 0.9774) compared to TRISS and other ML models in the prediction of patient mortality (37). A recent study from Servia et al. used data from the National Trauma Registry of 52 Spanish ICUs to test the predictive capabilities for mortality on nine ML-based classifiers on data from 9,790 critically injured patients, showing a high correlation of mortality in patients with traumatic brain injury and organic failure. Even though all tested classifiers were able to produce a high accuracy, specificity, and AUC, low values for recall were obtained. Servia et al. discussed that since comparable results in accuracy and sensitivity could be achieved by all nine classifiers, one should rather choose ML techniques by their architecture and fit to a specific task than by the determination of statistical superiority only (38).

## Discussion

Decision-making in the acute management of severely injured patients has to be based on reliable and accurate statements and predictions.

In the past, experience-based algorithms like TRISS have shown weaknesses in clinical use. Data-driven ML tools show great potential as a new approach to problems of this nature. In this systematic review, we could show that ML tools are generally more accurate and sensitive when compared to existing tools for decision-making in acute trauma management like GCS, ISS, and TRISS. Only one of eight studies that compared prediction performance showed TRISS superior when compared to the tested ML mechanisms.

Mortality was the most frequently predicted outcome parameter in our review (*n* = 19). We included this outcome even though the use of decision aid for predicting mortality is discussable. Using vital signs and BGA parameters, mortality can be predicted with higher accuracy and sensitivity compared to methods in clinical use right now. Different models, more recently ANNs, can solve the task of determining the mortality risk of a patient in the resuscitation bay, but the implementation of these tools assumes digitalized parameters of patients’ vital signs, lab parameters, and demographics at the time of hospitalization. Furthermore, interfaces need to be in place to make these predictions possible and actually aid in the acute management of trauma patients. We hypothesize that these are limitations to the broad implementation of these tools. Clinical validation of such tools also mandates standardization of evaluation parameters of ML models fit for clinical use. The benefit of mortality prediction in the acute setting of a resuscitation room remains unclear. However, if an implementation can aid in acute management and the above-mentioned limitations are addressed, we consider ML tools as a promising development. Would we do less if a patient had a 70% probability of 48 h-survival?

Because of the broad range of different trauma mechanisms, injury pattern prediction showed a heterogeneous field of studies that all predicted specific outcomes with different ML models. The complexity of the prediction makes it hard to generalize injury patterns of trauma patients in one ML model so far. Another field with similar limitations was the prediction of emergency interventions. One paper was able to predict the need for DCL with high sensitivity and specificity, but the often complex indication of different emergency or damage control interventions makes predictions on a general scale complicated. Further developments are needed to make ML models implementable for these tasks.

Also, hemorrhage and the subsequent need for transfusion can be predicted by different ML models; recent publications especially show high accuracy in these predictions. If the implementation of live evaluation of vital signs and BGA parameters is widely available, ML-powered decision support for transfusion protocols seems likely to be implemented in the near future. Only one article set the prediction of ICU length of stay as the main outcome of ANN implementation and was able to show that the prediction made by the network did not significantly differ from the real length of stay after training on 95 cases. However, due to the small number of cases and lack of comparable studies, we cannot assess the validity of these findings yet.

Specific problems cannot always be answered best by one ML model or approach. To evaluate models only on their differences in accuracy or sensitivity does not always reflect their implementation ability, especially in a clinical environment, with the need for clinical evaluation and standardization.

ANNs show a wide range of implementation possibilities for the future but are very sparsely implemented in the clinical routine so far. To our knowledge, none of the shown ML tools in the included studies seem to be in actual clinical use in the version described in the respective articles. One reason is the need for big data to train these ML tools, reflected by the large number of studies in this systematic review (10 of 36) training and testing their models on data from national or international registries or databases. Unfortunately, data generation in such databases can be incomplete at times and are not standardized internationally. Subsequently, there will be a call for access to well-structured and complete datasets for trauma patients in the future, which we hope will enable the training of generalized ML and ANN models.

## Conclusion

Digitalization and general computing and technical capabilities of healthcare providers are the basis on which implementation of ML models into the clinical routine can be made possible. While the benefits of these ML tools, especially ANNs for prediction in different fields (image segmentation, image processing) are undeniable, several requirements concerning live availability of data, better and more accessible big data sets on trauma patients, technical requirements on site, and insurance of patient data security need to be met before the implementation of ML techniques, and especially ANNs can become a widely implemented reality in the acute management of trauma patients.

## Data Availability

The original contributions presented in the study are included in the article/Supplementary Material, further inquiries can be directed to the corresponding author/s.

## References

[1] Berkeveld E Popal Z Schober P Zuidema WP Bloemers FW Giannakopoulos GF. Prehospital time and mortality in polytrauma patients: a retrospective analysis. BMC Emerg Med. (2021) 21:78. 10.1186/s12873-021-00476-6 34229629 PMC8261943

[2] Hussmann B Lendemans S. Pre-hospital and early in-hospital management of severe injuries: changes and trends. Injury. (2014) 45(Suppl 3):S39–42. 10.1016/j.injury.2014.08.016 25284232

[3] Park Y Lee GJ Lee MA Choi KK Gwak J Hyun SY Major causes of preventable death in trauma patients. J Trauma Inj. (2021) 34:225–32. 10.20408/jti.2020.0074

[4] Spanjersberg WR Bergs EA Mushkudiani N Klimek M Schipper IB. Protocol compliance and time management in blunt trauma resuscitation. Emerg Med J. (2009) 26:23–7. 10.1136/emj.2008.058073 19104091

[5] Topol EJ. High-performance medicine: the convergence of human and artificial intelligence. Nat Med. (2019) 25:44–56. 10.1038/s41591-018-0300-7 30617339

[6] Deo RC. Machine learning in medicine. Circulation. (2015) 132:1920–30. 10.1161/CIRCULATIONAHA.115.001593 26572668 PMC5831252

[7] Moher D Liberati A Tetzlaff J Altman DG. Preferred reporting items for systematic reviews and meta-analyses: the PRISMA statement. PLoS Med. (2009) 6:e1000097. 10.1371/journal.pmed.1000097 19621072 PMC2707599

[8] Ogunyemi OI Clarke JR Ash N Webber BL Combining geometric and probabilistic reasoning for computer-based penetrating-trauma assessment. J Am Med Inform Assoc. (2002) 9:273–82. 10.1197/jamia.m0979 11971888 PMC344587

[9] Metzger M Howard M Kellogg L Kundi R. Ensemble prediction of vascular injury in trauma care initial efforts towards data-driven, low-cost screening. Proceedings 2015 IEEE International Conference on Big Data:2560–8 (2015).

[10] Chen L Ogundele O Clermont G Hravnak M Pinsky MR Dubrawski AW. Dynamic and personalized risk forecast in step-down units. Implications for monitoring paradigms. Ann Am Thorac Soc. (2017) 14:384–91. 10.1513/AnnalsATS.201611-905OC 28033032 PMC5427723

[11] Paydar S Parva E Ghahramani Z Pourahmad S Shayan L Mohammadkarimi V Do clinical and paraclinical findings have the power to predict the critical conditions of injured patients after traumatic injury resuscitation? Using data mining artificial intelligence. Chin J Traumatol. (2020) 24(1):48–52. 10.1016/j.cjtee.2020.11.009 33358634 PMC7878456

[12] Kulshrestha S Dligach D Joyce C Baker MS Gonzalez R O'Rourke AP Prediction of severe chest injury using natural language processing from the electronic health record. Injury. (2020) 52(2):205–12. 10.1016/j.injury.2020.10.094 33131794 PMC7856032

[13] Walczak S. Artificial neural network medical decision support tool: predicting transfusion requirements of ER patients. IEEE Trans Inf Technol Biomed. (2005) 9:468–74. 10.1109/titb.2005.847510 16167701

[14] Chen L McKenna TM Reisner AT Gribok A Reifman J. Decision tool for the early diagnosis of trauma patient hypovolemia. J Biomed Inform. (2008) 41:469–78. 10.1016/j.jbi.2007.12.002 18255342

[15] Hodgman EI Cripps MW Mina MJ Bulger EM Schreiber MA Brasel KJ External validation of a smartphone app model to predict the need for massive transfusion using five different definitions. J Trauma Acute Care Surg. (2018) 84:397–402. 10.1097/TA.0000000000001756 29200079 PMC5780249

[16] Christie SA Conroy AS Callcut RA Hubbard AE Cohen MJ. Dynamic multi-outcome prediction after injury: applying adaptive machine learning for precision medicine in trauma. PLoS One. (2019) 14:e0213836. 10.1371/journal.pone.0213836 30970030 PMC6457612

[17] Bhat A Podstawczyk D Walther BK Aggas JR Machado-Aranda D Ward KR Toward a hemorrhagic trauma severity score: fusing five physiological biomarkers. J Transl Med. (2020) 18:348. 10.1186/s12967-020-02516-4 32928219 PMC7490913

[18] Clarke JR Hayward CZ Santora TA Wagner DK Webber BL. Computer-generated trauma management plans: comparison with actual care. World J Surg. (2002) 26:536–8. 10.1007/s00268-001-0263-5 12098040

[19] Hirshberg A Wall MJ Jr Mattox KL. Bullet trajectory predicts the need for damage control: an artificial neural network model. J Trauma. (2002) 52:852–8. 10.1097/00005373-200205000-00006 11988649

[20] Harvin JA Kao LS Liang MK Adams SD McNutt MK Love JD Decreasing the use of damage control laparotomy in trauma: a quality improvement project. J Am Coll Surg. (2017) 225:200–9. 10.1016/j.jamcollsurg.2017.04.010 28445796 PMC5533621

[21] Wolfe R McKenzie DP Black J Simpson P Gabbe BJ Cameron PA. Models developed by three techniques did not achieve acceptable prediction of binary trauma outcomes. J Clin Epidemiol. (2006) 59:26–35. 10.1016/j.jclinepi.2005.05.007 16360558

[22] Liu NT Holcomb JB Wade CE Darrah MI Salinas J. Utility of vital signs, heart rate variability and complexity, and machine learning for identifying the need for life-saving interventions in trauma patients. Shock. (2014) 42:108–14. 10.1097/SHK.0000000000000186 24727872

[23] Liu NT Holcomb JB Wade CE Batchinsky AI Cancio LC Darrah MI Development and validation of a machine learning algorithm and hybrid system to predict the need for life-saving interventions in trauma patients. Med Biol Eng Comput. (2014) 52:193–203. 10.1007/s11517-013-1130-x 24263362

[24] Gholipour C Rahim F Fakhree A Ziapour B. Using an artificial neural networks (ANNs) model for prediction of intensive care unit (ICU) outcome and length of stay at hospital in traumatic patients. J Clin Diagn Res. (2015) 9:OC19–23. 10.7860/JCDR/2015/9467.5828 26023581 PMC4437096

[25] Ahmed FS Ali L Joseph BA Ikram A Ul Mustafa R Bukhari SAM. A statistically rigorous deep neural network approach to predict mortality in trauma patients admitted to the intensive care unit. J Trauma Acute Care Surg. (2020) 89:736–42. 10.1097/TA.0000000000002888 32773672

[26] Almaghrabi F Xu D-L Yang J-B. A new machine learning technique for predicting traumatic injuries outcomes based on the vital signs. 2019 25th IEEE International Conference on Automation and Computing (Icac):64–8 (2019).

[27] DiRusso SM Chahine A Sullivan T Cuff S Nealon P Siegel B Development of an artificial neural network to predict trauma survival in pediatric patients. Crit Care Med. (2000) 28:A29.

[28] Fuller JJ Emmett M Kessel JW Price PD Forsythe JH. A comparison of neural networks for computing predicted probability of survival for trauma victims. W V Med J. (2005) 101:120–5. PMID: 16161530

[29] Gorczyca MT Toscano NC Cheng JD. The trauma severity model: an ensemble machine learning approach to risk prediction. Comput Biol Med. (2019) 108:9–19. 10.1016/j.compbiomed.2019.02.025 30965177

[30] Gorczyca MT Toscano NC Cheng JD. Time-dependent prediction and evaluation of variable importance using superlearning in high-dimensional clinical data. J Trauma Acute Care Surg. (2013) 75:S53–60. 10.1097/TA.0b013e3182914553 23778512 PMC3744063

[31] Kim D Cogill S Yang S. A data-driven artificial intelligence model for remote triage in the prehospital environment. PLoS One. (2018) 13:e0206006. 10.1371/journal.pone.0206006 30352077 PMC6198975

[32] Pearl A Caspi R Bar-Or D. Artificial neural network versus subjective scoring in predicting mortality in trauma patients. Stud Health Technol Inform. (2006) 124:1019–24. PMID: 17108643

[33] Pearl A Bar-Or R Bar-Or D. An artificial neural network derived trauma outcome prediction score as an aid to triage for non-clinicians. Stud Health Technol Inform. (2008) 136:253–8 PMID: 18487740

[34] Rau C-S Wu S-C Chuang J-F Huang C-Y Liu H-T Chien PC Machine learning models of survival prediction in trauma patients. J Clin Med. (2019) 8:799. 10.3390/jcm8060799 PMC6616432 31195670

[35] Roveda G Koledoye MA Parimbelli E Holmes JH. Predicting clinical Outcomes in Patients With Traumatic Bleeding: A Secondary Analysis of the CRASH-2 Dataset. 2017 IEEE 3rd International Forum on Research and Technologies for Society and Industry (Rtsi):332–7 (2017).

[36] Schetinin V Jakaite L Krzanowski W. Bayesian averaging over decision tree models for trauma severity scoring. Artif Intell Med. (2018) 84:139–45. 10.1016/j.artmed.2017.12.003 29275896

[37] Sefrioui I Amadini R Mauro J El Fallahi A Gabbrielli M. Survival prediction of trauma patients: a study on US National Trauma Data Bank. Eur J Trauma Emerg Surg. (2017) 43:805–22. 10.1007/s00068-016-0757-3 28229175

[38] Serviá L Montserrat N Badia M Llompart-Pou JA Barea-Mendoza JA Chico-Fernández M Machine learning techniques for mortality prediction in critical traumatic patients: anatomic and physiologic variables from the RETRAUCI study. BMC Med Res Methodol. (2020) 20:262. 10.1186/s12874-020-01151-3 33081694 PMC7576744

[39] Partridge D Schetinin V Li D Coats TJ Fieldsend JE Krzanowski WJ Interpretability of Bayesian decision trees induced from trauma data. Medical Image Computing and Computer Assisted Intervention - Miccai. (2006) 2019(Pt Iii 4029):972–81.

[40] Tsiklidis EJ Sims C Sinno T Diamond SL. Using the National Trauma Data Bank (NTDB) and machine learning to predict trauma patient mortality at admission. PLoS One. (2020) 15:e0242166. 10.1371/journal.pone.0242166 33201935 PMC7671512

[41] Schetinin V Jakaite L Krzanowski WJ. Prediction of survival probabilities with Bayesian decision trees. Expert Syst Appl. (2013) 40:5466–76. 10.1016/j.eswa.2013.04.009

